# Mitochondrial Genome Sequences Effectively Reveal the Phylogeny of *Hylobates* Gibbons

**DOI:** 10.1371/journal.pone.0014419

**Published:** 2010-12-23

**Authors:** Yi-Chiao Chan, Christian Roos, Miho Inoue-Murayama, Eiji Inoue, Chih-Chin Shih, Kurtis Jai-Chyi Pei, Linda Vigilant

**Affiliations:** 1 Department of Primatology, Max-Planck Institute for Evolutionary Anthropology, Leipzig, Germany; 2 Gene Bank of Primates and Primate Genetics Laboratory, German Primate Center, Göttingen, Germany; 3 Wildlife Research Center, Kyoto University, Kyoto, Japan; 4 Graduate School of Science, Kyoto University, Kyoto, Japan; 5 Animal Division, Taipei Zoo, Taipei, Taiwan; 6 Institute of Wildlife Conservation, National Pingtung University of Science and Technology, Pingtung, Taiwan; Smithsonian Institution National Zoological Park, United States of America

## Abstract

**Background:**

Uniquely among hominoids, gibbons exist as multiple geographically contiguous taxa exhibiting distinctive behavioral, morphological, and karyotypic characteristics. However, our understanding of the evolutionary relationships of the various gibbons, especially among *Hylobates* species, is still limited because previous studies used limited taxon sampling or short mitochondrial DNA (mtDNA) sequences. Here we use mtDNA genome sequences to reconstruct gibbon phylogenetic relationships and reveal the pattern and timing of divergence events in gibbon evolutionary history.

**Methodology/Principal Findings:**

We sequenced the mitochondrial genomes of 51 individuals representing 11 species belonging to three genera (*Hylobates*, *Nomascus* and *Symphalangus*) using the high-throughput 454 sequencing system with the parallel tagged sequencing approach. Three phylogenetic analyses (maximum likelihood, Bayesian analysis and neighbor-joining) depicted the gibbon phylogenetic relationships congruently and with strong support values. Most notably, we recover a well-supported phylogeny of the *Hylobates* gibbons. The estimation of divergence times using Bayesian analysis with relaxed clock model suggests a much more rapid speciation process in *Hylobates* than in *Nomascus*.

**Conclusions/Significance:**

Use of more than 15 kb sequences of the mitochondrial genome provided more informative and robust data than previous studies of short mitochondrial segments (e.g., control region or cytochrome b) as shown by the reliable reconstruction of divergence patterns among *Hylobates* gibbons. Moreover, molecular dating of the mitogenomic divergence times implied that biogeographic change during the last five million years may be a factor promoting the speciation of Sundaland animals, including *Hylobates* species.

## Introduction

**Figure 1:**
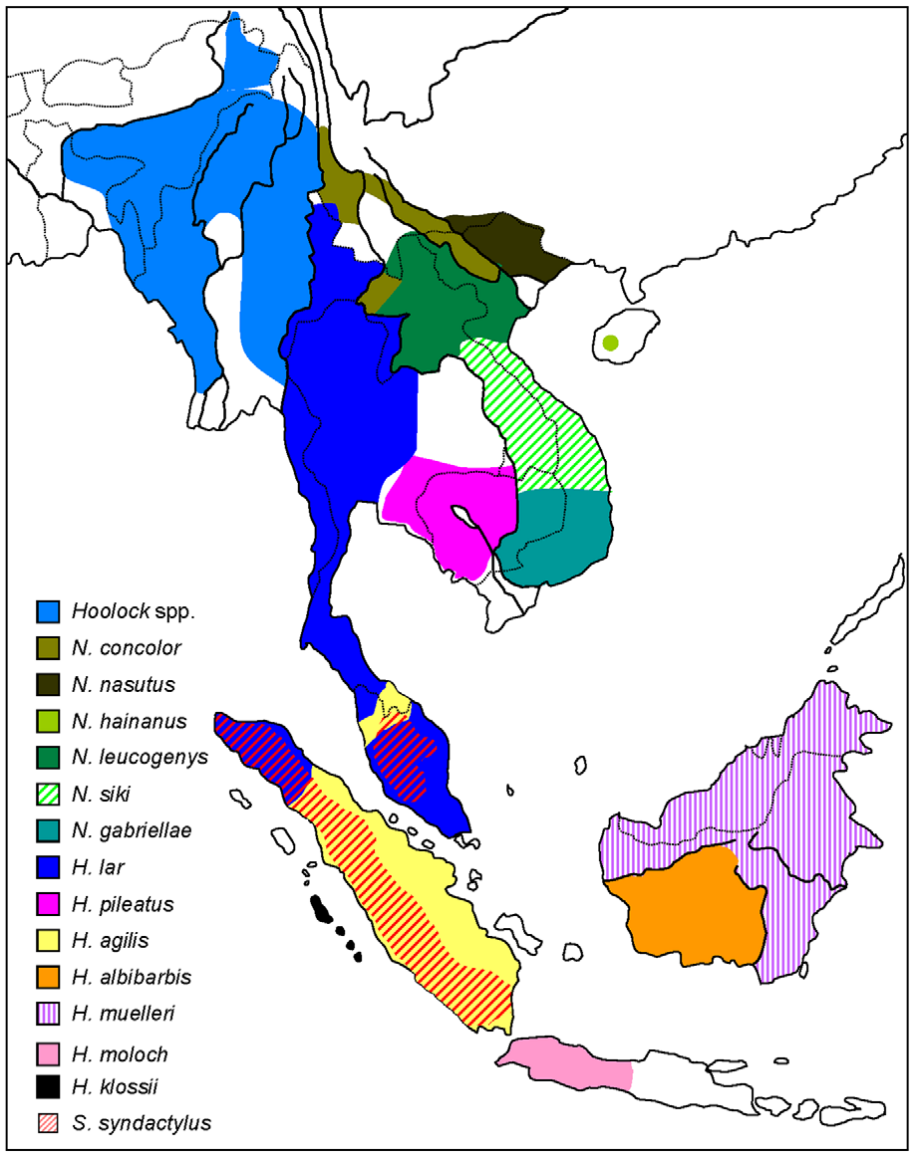
Approximate geographic distribution of gibbons. Dotted and solid lines indicate country borders and major rivers, respectively. Adapted from [4].

Gibbons (Hylobatidae) are small arboreal apes living in tropical and sub-tropical forests of the mainland and islands of Southeast Asia, including the Malay Peninsula, Sumatra, Borneo, Java and Mentawai Islands (Figure 1). Uniquely among contemporary hominoids, gibbons exist as multiple geographically contiguous taxon exhibiting distinctive behavioral, morphological, and karyotypic characteristics. Gibbons are classified into four genera *Hylobates*, *Hoolock*, *Nomascus*, and *Symphalangus*, each of which features a different number of chromosomes [1], [2], [3], [4], [5]. The number of species and subspecies is a more problematic issue, with some authorities listing as many as 28 potential taxa [2], while others limit themselves to 25 taxa and differ as to whether there may be 16 [5] or 18 species [4] therein. In contrast, two species of orangutans and gorillas have been recognized [1], [2] along with the bonobo and four subspecies of chimpanzees [1] meaning that the speciosity of gibbons provides a startling contrast to the situation in other ape lineages.

The evolutionary relationships of gibbon taxa have long been a focus of study due to their high taxonomic diversity and conservation importance, as nearly all gibbons have been classified as endangered at either the species or subspecies level [5]. Numerous morphological and molecular studies on the phylogenetic relationships among Hylobatidae members have led to consensus on the monophyly of each of the four genera [6], [7], [8], [9], [10]. A scenario in which *Nomascus* diverged first, followed by *Symphalangus*, *Hoolock* and then *Hylobates* is well-supported by analyses of mtDNA control region segments [6], and is also inferred, albeit with weaker statistical support, in a more recent study of mtDNA cytochrome b (cyt b) sequences [4]. While the genera *Hoolock* and *Symphalangus* contain just two and one species, respectively, some half-dozen species have been attributed to both *Nomascus* and *Hylobates*. Recent analysis of mtDNA cyt b sequences produced a statistically supported description of phylogenetic relationships among *Nomascus* spp. [4], [11]. In contrast, studies of short segments of mtDNA have proven unable to confidently resolve the phylogenetic relationships among the members of the genus *Hylobates* [4], [8], [10], [12], [13]. This suggests that while the evolutionary radiation of *Nomascus* gibbons appears to have occurred in a stepwise fashion over some 4 million years, in the case of *Hylobates* short mtDNA sequences (control region [10], cyt b [4], [12], ND3, ND4 and ND5 genes [8], [13] do not provide enough information for depicting their apparently rapid evolutionary radiation.

In addition to the pattern of evolutionary relationships, the timings of the molecular divergences within gibbons are also of interest. Results from mtDNA studies suggested that the split of great apes and gibbons occurred 15–20 million years ago (mya) [4], [14], [15], [16], [17]. Two studies relying on mtDNA cyt b sequences suggest that a rapid radiation of gibbons began some 8 to 10 mya [4], [18]. In particular, the differentiation of the species within the *Nomascus* genera was inferred to have occurred from 4.2 mya to as recently as 0.55 mya [4]. It would be interesting to compare the timings of the divergences within *Nomascus* to those within the similarly speciose *Hylobates* genus, but this is hampered by the lack of a reliable phylogeny for *Hylobates*.

Recent phylogenetic studies reveal that longer mtDNA sequences, e.g. mitochondrial genomes (mtgenomes), can provide sufficient resolution for reconstructing a robust phylogeny [19], [20], [21], [22], [23], [24], [25] and also facilitate the molecular dating of divergence events within a phylogeny [16], [17], [22], [23], [24], [25], [26]. Whole mtDNA genome sequences can be recovered efficiently by amplifying the entire ∼16 kb genome in one or two fragments, which also serves to reduce the chance of inadvertent analysis of segments of mtDNA that have translocated to the nuclear genome (‘numts’) [27]. Unfortunately, the amplification of such large segments of the mitochondrial genome demands high-quality DNA and is not successful when applied to the limited amount of degraded DNA obtained from non-invasively obtained materials such as hair or feces. Many gibbon taxa are present in very small populations and are not held in captivity, so that only non-invasive samples can be collected. For example, one recent study used DNAs from blood, tissues, feces and hair to achieve a very comprehensive sampling of gibbon taxa, but was necessarily limited to analysis of a small proportion of the mtDNA molecule and so not all inferences were supported with statistical confidence [4]. In contrast, researchers in another recent study generated whole mtDNA genome sequences from gibbons, but analyzed only five individuals representing three of the four genera, and thus although the topology was well-supported the insights were limited [15].

Here we sought to generate mitochondrial genome sequences from as many gibbons as possible in order to improve the resolution of the evolutionary relationships among members of the gibbon family and assess the timings of various divergence events. We generated DNA sequences of entire mitochondrial genomes from 51 gibbons representing three of the four genera and 11 different species. These data produce a reliable phylogeny featuring strong support values and most notably contribute to resolution of the phylogeny of *Hylobates* species and the timings of molecular divergence events.

## Results and Discussion

### Gibbon mitochondrial genome sequences

We produced whole mtDNA genome sequences from 51 individuals. From each individual we obtained an average of 1,965 tagged reads with an average length of 191 bp, thus yielding approximately 410 kb of sequence data corresponding to 24-fold coverage of the gibbon mitochondrial genome. We assembled tagged reads from each individual into consensus contigs within which sites with low coverage (<5-fold) and ambiguous sites were marked as missing data (N). Within the 51 consensus contigs the percentage of missing data ranged between 0 and 13.54%. The positions with missing data were further examined and found to mostly occur in the control region, so that for each individual the percentage of positions with missing data outside the control regions ranged from 0 to 8.84%.

The 51 mtgenome sequences represent 11 gibbon species belonging to three genera, *Hylobates*, *Nomascus* and *Symphalangus*. All gibbon mitochondrial genomes consisted of the 22 tRNA genes, 2 rRNA genes, 13 protein-coding genes and the control region in the order typically observed in vertebrates. In common with other studies generating and analyzing whole mtDNA sequences for interspecific comparisons (e.g. [23]) we noted that the control region tends to contain sites with missing data and because of its high rate of evolution can be difficult to align among species. Therefore, we produced concatenated sequences of the 37 individual genes for further analyses. These concatenated sequences ranged from 15,407–15,416 bp in length.

### Phylogenetic analyses

**Figure 2:**
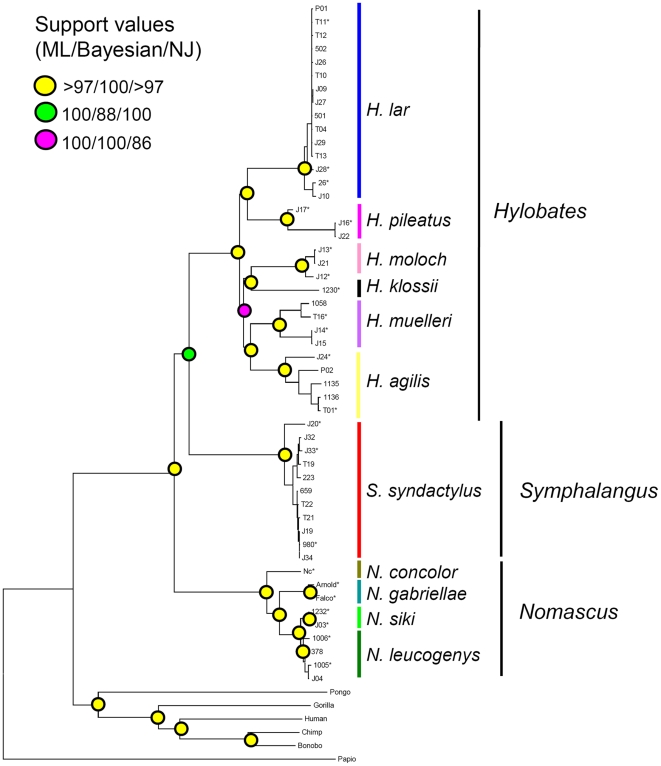
Phylogenetic tree of gibbons and outgroup primates based on the mtDNA concatenated dataset. The phylogenetic relationships among gibbons and six outgroup primates were inferred from the mtDNA concatenated dataset, including three partitioned sets: ribosomal RNA, transfer RNA and protein-coding gene. The maximum likelihood (ML), Bayesian and neighbor-joining (NJ) methods were used to reconstruct phylogenetic trees. All three analyses produced the same topology and their support values are indicated by circles on the nodes of the NJ tree shown here. Individuals used in the estimation of divergence times are marked with an asterisk.

All three methods used for phylogeny reconstructions produced the same topology with strong support values (Figure 2). The three gibbon genera examined here appear as monophyletic clades with *Nomascus* diverging first followed by *Symphalangus* and *Hylobates*.

Within *Nomascus*, each of the four species represented here forms a well-supported monophyletic clade or, as in the case of *N. concolor*, a distinct lineage. *N. concolor* is the basal taxon here, but we lack samples from *N. hainanus* and *N. nasutus* which form the earliest divergence in other recent work [4]. Following the divergence of *N. concolor* there is a divergence between *N. gabriellae* and the *N. leucogenys/N. siki* clade. *N. leucogenys* and *N. siki* are each monophyletic and exhibit a recent divergence from each other. These results are highly supported and entirely consistent with findings based upon analysis of the cyt b gene [4].

Our analysis of mtDNAs from six of seven species of *Hylobates* gibbons produces a well-supported phylogeny in which each species is represented by distinct lineages or monophyletic clades. The most closely related pairs of species consist of *H. lar* and *H. pileatus*, *H. klossii* and *H. moloch*, and *H. agilis* and *H. muelleri*. We lack samples from *H. alibarbis*, which would be expected to be closely related to *H. agilis* [4]. As is not unexpected, our *Hylobates* phylogeny differs from previous work that did not achieve statistical reliability [4], [8], [10], [12], [13]. These studies had relied on information from different mtDNA segments, e.g. control region [10], cyt b [4], [12], ND3, ND4 and ND5 genes [8], [13]. Use of only one of these segments provided too little information for confident resolution of a phylogeny featuring short branch lengths between divergences [8], [10], [22], [28], [29].

Unlike the other genera, the siamang (*Symphalangus*) only occurs as a single species, which is supported by the monophyly of the mtDNAs examined here.

### Estimation of divergence times

Using three fossil calibration points we estimated mtDNA divergences times within gibbons (Table 1). The divergence time between gibbons and great apes was estimated at 19.25 million years ago. This is compatible with the Bayesian estimations of other mtgenomic studies [15], [16], [17] and predates slightly the estimates of 16.26 mya based on the mtDNA cyt b gene [4].

**Table 1:** Bayesian estimates of divergence times based on the relaxed molecular clock approach.

Node	Divergence	mean	95% HPD
t1*	Baboon (*Papio*)-apes	22.96	21.01–24.82
t2	Great apes-Gibbons	19.25	15.54–22.99
t3	Gibbons (*Nomascus*-other gibbon spp.)	8.67	5.33–12.53
t4	*Symphalangus*-*Hylobates*	7.52	4.48–10.94
t5	*Hylobates* (2 *Hylobates* subclades)	4.17	2.48–6.13
t6	*H. lar*-*H. pileatus*	2.90	1.45–4.5
t7	2 *Hylobates* sister groups (*H. moloch*/*H.klossii*-*H. muelleri*/*H. agilis*)	3.45	2.04–5.1
t8	*H. moloch*-*H. klossii*	2.77	1.47–4.19
t9	*H. muelleri*-*H. agilis*	2.62	1.47–4
t10	*N. concolor*-other *Nomascus* spp.	2.37	1.07–4
t11	*N. gabriellae*- *N. leucogenys*/*N. siki*	1.40	0.57–2.44
t12	*N. leucogenys*-*N. siki*	0.46	0.19–0.82
t13	MRCA *Symphalangus*	0.81	0.31–1.53
t14	MRCA *H. lar*	0.50	0.19–0.91
t15	MRCA *H. pileatus*	0.71	0.35–1.15
t16	MRCA *H. moloch*	0.40	0.11–0.79
t17	MRCA *H. muelleri*	1.24	0.47–2.19
t18	MRCA *H. agilis*	0.92	0.36–1.66
t19	MRCA *N. gabriellae*	0.21	0.06–0.41
t20	MRCA *N. siki*	0.06	0.01–0.12
t21	MRCA *N. leucogenys*	0.26	0.08–0.49
t22*	Great apes (orangutan-other great apes)	14.02	12.24–15.89
t23	Gorilla-Human/*Pan*	8.95	6.95–11.08
t24*	Human-*Pan*	6.35	5.41–7.26
t25	*Pan* (chimpanzee-bonobo)	2.38	0.94–4.04

The divergence time estimates are in million years before present. MRCA denotes the most recent common ancestor. HPD, highest posterior density.

*Nodes used for calibration.

Although we could not sample representatives of the genus *Hoolock*, our inference of the relationships among genera is otherwise similar to the well-supported phylogeny of genera presented by Roos and Geissmann [6], in which *Hoolock* diverges after *Symphalangus* but before *Hylobates*. Thus asssuming that the initial split within gibbons occurred between *Nomascus* and all remaining gibbons, we infer a date for this event of around 8.67 mya (for 95% confidence intervals see Table 1), in comparison with a recent estimate of 8.34 mya based on cyt b sequences [4]. The divergences among the four *Nomascus* species examined here began at around 2.4 mya. However, this is likely an underestimate as in comparison with the recent work by Thinh and coworkers we lack samples from *N. hainanus* and *N. nasutus*, which would be expected to form an early divergence in *Nomascus* that has been dated to 4.24 mya [4]. Our results concur in estimating the divergence of *N. gabriellae* at 1.40 mya (1.74 mya in [4]). We find that the most recent species split of *Nomascus* gibbons occurred between *N. leucogenys* and *N. siki* at 0.46 mya, consistent with the date of 0.55 mya previously inferred [4]. In sum, divergence events within *Nomascus* appear to have occurred over a relatively long period of time, resulting in the appearance of some six species in more than 4 million years.

In contrast to the apparent stepwise fashion of divergences in *Nomascus*, the radiation of *Hylobates* gibbons began with an initial divergence at 4.17 mya, followed by a split between *H. lar* and *H. pileatus* at 2.90 mya and nearly contemporaneous molecular divergences between two additional species pairs (*H. moloch* and *H. klossii*, *H. muelleri* and *H. agilis*) at 2.77 and 2.62 mya, respectively. In contrast to the successive divergences of the *Nomascus* species, the species radiation within *Hylobates* occurred over a much shorter time period of only 1.5 million years, thus explaining the need for larger datasets to resolve the divergences within the genus with significance. Consistent with other studies, we infer that the radiation within *Hylobates* began at about 4 mya [4], [15].

There are three pairs of *Hylobates* species which come into contact and reportedly hybridize in sympatry. These species include *H. albibarbis* and *H muelleri*, *H agilis* and *H. lar*, and *H. lar* and *H. pileatus* [30]. In our study, all examined *Hylobates* species with multiple samples were monophyletic in the phylogenetic analyses, suggesting that no individuals were representative of recent female-mediated gene flow between species. Studies of multiple independent genetic markers, ideally using samples of known geographic provenance, would be needed to effectively address the question of the extent and consequences of gene flow among *Hylobates* species.

### Broader conclusions

Using mtDNA genome sequences to analyze the phylogenetic relationships of 11 extant gibbon species belonging to three genera, we obtained a phylogeny characterized by confident support values of all nodes and most noteworthily, among *Hylobates* species. We showed that complete mtgenome sequences provide sufficient information to resolve the sequence of events in the rapid divergence of *Hylobates*. The Bayesian inferences of split times were consistent with previous work. However, it is interesting to note that the confidence intervals surrounding those divergence time estimates are still rather large (Table 1). Although our divergence time estimates are generally similar to those obtained by other researchers using smaller mtDNA segments (e.g. cyt b [4]), the confidence intervals do not appear to have narrowed despite the use of this larger dataset. For example, the width of the confidence interval around the estimated 19.25 mya great ape and gibbon divergence is 7.45 my, even larger than the 3.47 my interval around the estimate of 16.26 mya previously estimated [4]. Since we used the same fossil-based calibration points as previous researchers and identical settings for the analysis, this result is most likely attributable to the difficulties inherent in modeling the inherently heterogeneous patterns of mutation of various mtDNA protein-coding genes.

We estimated that the timings of most splitting events in the Hylobatidae family occurred from the late Miocene to the Pliocene (11.6-2.6 mya), as was also observed in other diverse mammalian families, including bears (the Ursidae, [21]), modern cats (the Felidae, [31]), deer (the Cervidae, [32]), and true seals (the Phocidae, [33]). The coincidence of speciation times across different families implied that environmental changes around that time may have played an essential role in the evolutionary processes of gibbons and other mammals [34], [35], [36]. Of all gibbon genera, *Hylobates* has the largest distribution spreading throughout the Sundaland, which is characterized by high levels of species richness and endemism (biodiversity hotspot, [37]). The divergence events within the *Hylobates* phylogeny were estimated to have occurred in the Pliocene (5.3-2.6 mya). During this period, speciation events were also reported for other Sundaland animals, e.g. mutualistic *Crematogaster* ants [38], *Ficedula* flycatchers [39] and *Sundasciurus* tree squirrels [40]. We suggest that biogeographic changes in Southeast Asia in the past five million years, particularly those induced by sea level changes [41], may have been a factor promoting the evolutionary divergence of terrestrial animals in the Sundaland.

## Materials and Methods

### DNA samples and whole genome amplification

A total of 51 high-quality genomic DNA samples representing 11 species were collected (Table 2). All DNA samples used derive from long-term sample collections of the authors and were not acquired specifically for this study. These samples were previously collected from captive gibbons during routine veterinary care. All genomic DNA samples underwent a whole genome amplification (WGA) based upon the multiple displacement amplification procedure using GenomiPhi HY DNA Amplification Kit (GE Healthcare) and were purified by ethanol precipitation following the manufacturer's instructions. The purified WGA products were quantified on a Nanodrop spectrophotometer and used as templates for subsequent long-range PCRs for the amplification of the complete mtgenome.

**Table 2:** Gibbon samples used in the present study.

Genus	Species	onwer's ID/Barcode ID	working ID	origin^a^	current deposition^a^
*Hylobates*	*agilis*	20050292D10	T01	Taipei Zoo	Taipei Zoo
		1135	1135	Bristol Zoo	German Primate Center
		1136	1136	Bristol Zoo	German Primate Center
		941006G01	P02	Pingtung Rescue Center	Pingtung Rescue Center
		1061	J24	Ragunan Zoo	WRC, Kyoto University
	*lar*	20040082D10	T04	Taipei Zoo	Taipei Zoo
		20040113D10	T10	Taipei Zoo	Taipei Zoo
		20040284D10	T11	Taipei Zoo	Taipei Zoo
		20040285D10	T12	Taipei Zoo	Taipei Zoo
		20040286D10	T13	Taipei Zoo	Taipei Zoo
		960530G01	P01	Pingtung Rescue Center	Pingtung Rescue Center
		26	26	Wuppertal Zoo	German Primate Center
		501	501	Nuremberg Zoo	German Primate Center
		502	502	Nuremberg Zoo	German Primate Center
		2356	J09	Dusit Zoo	WRC, Kyoto University
		2357	J10	Dusit Zoo	WRC, Kyoto University
		1981	J26	Chiang Mai Zoo	WRC, Kyoto University
		1982	J27	Chiang Mai Zoo	WRC, Kyoto University
		2845	J28	PRI, Kyoto University	WRC, Kyoto University
		3400	J29	PRI, Kyoto University	WRC, Kyoto University
	*muelleri*	20050386D10	T16	Taipei Zoo	Taipei Zoo
		2520	J14	Kalimantan Samarinda	WRC, Kyoto University
		2521	J15	Kalimantan Samarinda	WRC, Kyoto University
		1058	1058	Rostock Zoo	German Primate Center
	*klossii*	1230	1230	Twycross Zoo	German Primate Center
	*moloch*	2486	J12	Ragunan Zoo	WRC, Kyoto University
		2488	J13	Ragunan Zoo	WRC, Kyoto University
		2349	J21	Ragunan Zoo	WRC, Kyoto University
	*pileatus*	2360	J16	Dusit Zoo	WRC, Kyoto University
		2361	J17	Dusit Zoo	WRC, Kyoto University
		2362	J22	Dusit Zoo	WRC, Kyoto University
*Symphalangus*	*syndactylus*	20050331D10	T19	Taipei Zoo	Taipei Zoo
		20060592D10	T21	Taipei Zoo	Taipei Zoo
		20060392D10	T22	Taipei Zoo	Taipei Zoo
		223	223	Munich Zoo	German Primate Center
		659	659	La Vallee des Singes	German Primate Center
		980	980	Krefeld Zoo	German Primate Center
		2506	J19	Ragunan Zoo	WRC, Kyoto University
		2507	J20	Ragunan Zoo	WRC, Kyoto University
		2512	J32	unknown	WRC, Kyoto University
		1973	J33	Padang, Sumatra, Indonesia	WRC, Kyoto University
		2506	J34	unknown	WRC, Kyoto University
*Nomascus*	*leucogenys*	378	378	Twycross Zoo	German Primate Center
		1005	1005	Duisburg Zoo	German Primate Center
		1006	1006	Duisburg Zoo	German Primate Center
		2364	J04	Dusit Zoo	WRC, Kyoto University
	*siki*	1232	1232	London Zoo	German Primate Center
		1986	J03	Chiangmai Zoo	WRC, Kyoto University
	*gabriellae*	Arnold	Arnold	Leipzig Zoo	MPI-EVA
		Falco	Falco	Leipzig Zoo	MPI-EVA
	*concolor*	1231	Nc	Twycross Zoo	German Primate Center

a Abbreviations: PRI, Primate Research Institute; WRC, Wildlife Research Center; MPI-EVA, Max-Planck Institute for Evolutionary Anthropology.

### Two-step multiplex long-range PCR

We amplified the entire mtgenomes in four large overlapping fragments (∼3.8–5 kb each) by two-step multiplex long-range PCR using the Expand Long Range dNTPack kit (Roche). In the first multiplex step, the four primer pairs were divided into two sets, A and B (Table 3). Each set contains two pairs of primers (the A set: pairs 1×12 and 6×9, the B set: pairs 4×10 and 5×11) and was used to amplify two non-overlapping fragments differing by ∼1 kb. Primers were designed by K. Finstermeier and M. Meyer to function across a wide range of primates and primers modified for use in gibbons were also used, as indicated. This step was performed in a total volume of 50 µl containing 100 ng of purified WGA product, 0.3 µM each primer, 5X buffer with MgCl_2_, 0.5 mM each dNTP and 3.5 unit of the Expand Long Range Enzyme mix under the following cycling conditions: initial denaturation at 92°C for 2 min; 9 cycles of 10 s at 92°C, 15 s at 57°C and 8 min at 68°C; then 21 cycles of 10 s at 92°C, 15 s at 57°C and 8 min at 68°C increasing by 20 s each cycle and a final elongation step of 15 min at 68°C. The PCR products of the first step were diluted 20-fold in water and 5 µl were used as templates in second step singleplex reactions using the same primer pairs as in the multiplex step. For example, we used diluted PCR products amplified with the set A as template to amplify the products of 4.1 kb using the pair 1×12 (primat_mt1_r and gibbon_mt12-2_f) in the singleplex reaction. The PCR conditions of the second step were the same as in the first, except that the final concentrations of the individual primer pairs was 3 µM and annealing temperature was 59°C. The singleplex PCR products were gel-cut and purified using QIAquick Gel Extraction Kit (Qiagen). The long-range PCR procedure enabled us to reduce the likelihood of amplifying possible nuclear mitochondrial pseudogenes (numts) [27], [42], [43], [44].

**Table 3:** Primers used for amplification of the entire mitochondrial genome.

Multiplex PCR sets	Singleplex PCR pairs	primer name	primer sequence (5′-3′)	expected product size^a^
**A**	1×12	primat_mt1_r	TGTCCTGATCCAACATCGAG	4.1 kb
		gibbon_mt12-2_f	CACGARACRGGATCAAACAAY	
	6×9	gibbon_mt6-2_r	GGAYCAGGTGACGAAYAGTGC	5.2 kb
		gibbon_mt9_f	AGGAAGGAATCGAACCCYC	
		gibbon_mt9-3_f^b^	ACCTTCTTYCCACAACACTTCC	
**B**	4×10	primat_mt4_f	CCGTGCAAAGGTAGCATAATC	4.9 kb
		gibbon_mt10_r	TATGGGGCTGGCTTGAAAC	
	5×11	primat_mt5_f	GGCTTTCTCAACTTTTAAAGGATA	3.8 kb
		primat_mt11_r	AGAATKYCAGYTTTGGGTRYTG	

a The approximate expected lengths of the singleplex PCR products.

b The primer gibbon_mt9-3_f was specific for amplification of the *N. concolor* sample.

### Sequencing of mitochondrial genomes

We used the high-throughput 454 sequencing technology with the parallel tagged sequencing (PTS) approach to sequence the gibbon entire mtgenome. The detailed protocols for preparation are described in Meyer et al. [45], [46] and the manufacturer's instructions (GS FLX platform, Roche). In brief, the purified PCR products were pooled by individual in equimolar ratios and sheared. Barcoding adapters with individual-specific tag sequences were ligated to the DNA fragments in each individual pool. A total of 51 unique tags were assigned to the 51 gibbon individuals, respectively. The individual tagged samples were then pooled together in equimolar ratios to produce the sequencing library in which the 454 adaptors were ligated to the tagged fragments. The library was quantified by quantitative PCR and used subsequently in the standard GS FLX sequencing procedure.

The 454 read sequence data were first sorted according to their tag sequences and then classified into the 51 subsets (51 individuals). The reads from each subset were assembled by MIA (Mapping Iterativ Assembler, http://sourceforge.net/projects/mia-assembler/) to create a consensus contig using the mtgenome of *H. lar* (GenBank X99256.1) as the reference sequence. The assembled consensus contig of each subset resulted in the mtgenome sequence of each gibbon individual.

### Sequence data analyses

The newly obtained mtgenome sequences were aligned with the *H. lar* reference using ClustalW 2.0 ([47], http://www.ch.embnet.org/software/ClustalW-XXL.html). The locations of rRNA, tRNA and protein-coding genes were determined by the comparison with the reference and the 37 individual genes were concatenated using DnaSP 5 [48]. To check for the presence of sequences derived from numts, the sequences of protein-coding genes were examined for frameshift or stop mutations and the rRNA sequences were compared with the known siamang rRNA numt (AF420053, [49]). No numt or other anomalous sequences were observed. The control region was excluded from the analyses. The mtgenome sequences of five great apes and one baboon were used as outgroups: *Pongo pygmaeus* (GenBank NC_001646), *Gorilla gorilla* (GenBank NC_001645), *Pan troglodytes* (GenBank NC_001643), *Pan paniscus* (GenBank NC_001644), *Homo sapiens* (GenBank AF347008) and *Papio hamadryas* (GenBank Y18001). The outgoup mtgenomes were processed using BioEdit 7.0.5 [50] to isolate their individual genes and to remove the control regions. The individual genes of outgroups were also concatenated with DnaSP 5. All obtained mtgenome sequences have been deposited in Genbank (Accession numbers HQ622758-HQ622808).

### Phylogenetic analyses

We aligned the concatenated sequences from gibbons and outgroup species using ClustalW. The best-fit nucleotide substitution model was selected by Model-Generator 0.85 [51] and the general time reversible (GTR) + I + Γ model was suggested. We partitioned the 15,484 bp alignment of the concatenated sequences into three schemes comprising (1) 12S and 16S rRNA genes combined, rRNA set (2) all 22 tRNA genes combined, tRNA set and (3) all 13 protein-coding genes combined, protein set. The information of alignment length, invariable, variable and informative sites for each scheme is listed in Table 4. We used this partitioned dataset for phylogeny reconstruction applying maximum likelihood (ML), Bayesian and neighbor-joining (NJ) methods as follows. We employed ML in RAxML 7.2.3 ([52], [53], http://phylobench.vital-it.ch/raxml-bb/index.php) with GTR + I + Γ substitution model to each partition. Bootstrap support values were based on 100 replicates. The Bayesian analysis was performed in MrBayes 3.1.2 ([54], http://cbsuapps.tc.cornell.edu/mrbayes.aspx). Four Metropolis-coupled Markov chain Monte Carlo (MCMC) analyses were run twice for 5,000,000 generations and sampled every 100 generations (mcmcp ngen = 5000000, nchains = 4, temp = 0.01, samplefreq = 100, burnin = 5000). The GTR + I + Γ substitution model was assigned to each partition. The NJ analysis with the bootstrap test was performed in MEGA 4.0 [55] using pairwise deletion for gaps/missing data. However, due to the unavailability of data partitioning and the GTR model in MEGA, the non-partitioned dataset and the best available Tamura-Nei model [56] were used for the NJ reconstruction.

**Table 4:** Sizes and characteristics of the partitioned datasets.

Schemes	rRNA	tRNA	Protein
Alignment length (bp)	2,552	1,530	11,402
No. of invariable sites	1,135	404	4,475
No. of variable sites	488	149	3,255
No. of informative sites	326	83	2,460

### Estimation of divergence times

We estimated the divergence times within the gibbon family using the Bayesian method implemented in the program BEAST 1.5.2 ([57], http://cbsuapps.tc.cornell.edu/beast.aspx) with a relaxed molecular clock approach [58]. Three fossil-based calibration points were applied: the split of hominoids-cercopithecoids (∼23 mya ±2 mya, [59], [60]), the separation of *Pongo* from the *Homo/Pan* lineage (∼14 mya ±1 mya, [61]) and the divergence between *Homo* and *Pan* (∼6.5 mya ±0.5 mya, [62], [63], [64]). Due to the high computational time demand for large datasets in BEAST, we used a sub-dataset of the protein set for analysis instead. In this sub-dataset, 22 gibbons (marked with stars in Figure 2) that are most divergent in their own species lineage and the six outgroup species are included. We partitioned this protein sub-dataset by codon positions (2 partitions: codon positions [1+2], 3) and unlinked the substitution model, rate heterogeneity and base frequencies across them. The tree topology obtained from above-mentioned phylogenetic analyses and the GTR + I + Γ substitution model were implemented in BEAST with the following settings: an uncorrelated lognormal relaxed clock model, Yule speciation process in tree prior, and 50,000,000 generations of MCMC steps sampled every 5000 generations. Two independent BEAST runs were carried out and the log output files were combined using LogCombiner 1.5.3 [57]. The effective sample size (ESS) values (greater than 380) were adequate for all parameters. Convergence was assessed in Tracer 1.5 (http://treebioedacuk/software/tracer/) and the first 1000 samples (5,000,000 generations) were excluded as burn-in. The chronological tree files were analyzed and visualized using TreeAnnotator 1.5.3 [57] and FigTree 1.3.1 (http://tree.bio.ed.ac.uk/software/figtree/).
