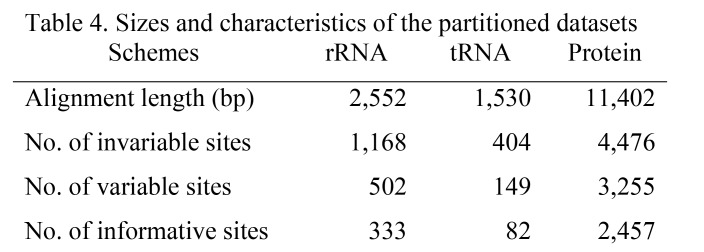# Correction: Mitochondrial Genome Sequences Effectively Reveal the Phylogeny of *Hylobates* Gibbons

**DOI:** 10.1371/annotation/9ca325c6-7a42-4cfd-9085-125571708b5c

**Published:** 2013-02-25

**Authors:** Yi-Chiao Chan, Christian Roos, Miho Inoue-Murayama, Eiji Inoue, Chih-Chin Shih, Kurtis Jai-Chyi Pei, Linda Vigilant

In regards to the error in Figure 2, the authors write:

"In this study, we generated and analyzed 51 mitochondrial genome (mtgenome) sequences of gibbons to reconstruct phylogenetic trees and estimate the divergence times in gibbon evolutionary history. We became recently aware that the sequences of two individuals (J24 and J17) in our dataset of 51 gibbon mtgenomes are partially incorrect and hence the corresponding Genbank records of these two sequences (accession numbers HQ622760 and HQ622786) have been removed from the GenBank database. Here we show the consequences of removing those two sequences from the analysis by examining newly generated phylogenetic trees and Bayesian estimates of divergence times.

Using the 49 mtgenome sequences, we reconstructed the phylogenetic trees of gibbons using Bayesian and maximum likelihood methods with three partitions (rRNA, tRNA and protein). The revised information on size and characteristics of the partitions are slightly different from those in the previous table (Table 4). While the branching pattern of the gibbon genera remain unchanged, the corrected results place *H. pileatus* in the basal position in the *Hylobates* phylogeny in contrast to the absence of basal species suggested in the previous results (Figure 2). The divergence times were newly estimated with a Bayesian relaxed clock approach using the protein sequences of mtgenomes from 22 individuals (indicated in the revised Figure 2), but in which the three tree priors of the previously observed monophylies (*H. lar-H. pileatus, H. klossii-H. moloch* and *H. agilis-H. muelleri*) were no longer set. We obtained somewhat younger estimates with the narrower intervals of 95% highest poster density (HPD) for dating the splitting events compared to the older estimates resulting from the previous analysis (Table 1). Namely, the results of corrected Bayesian estimation suggested that the timings of most splitting events in the Hylobatidae family occurred from the Late Miocene to the Early Pleistocene (11.61-0.78 million years ago) and the *Hylobates* radiation began some 3.32 million years ago.

Therefore, accordingly, we would like to replace the Table 4 of partition characteristics with a new revised table, and replace the Figure 2 of phylogenetic tree with a new revised figure as well. Moreover, a new table of corrected Bayesian estimates is also provided to replace the previous Table 1."

The correct Figure 2 can be seen here: 

**Figure:**
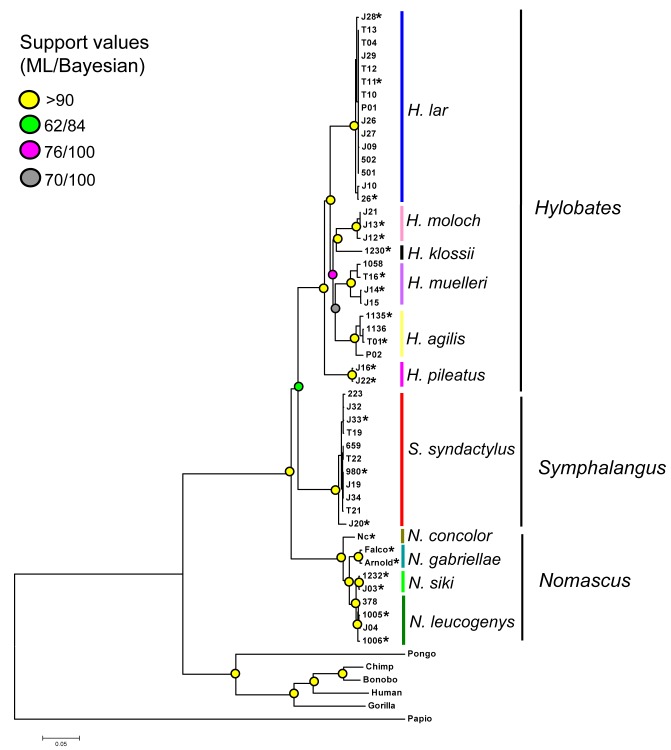


The correct legend for Figure 2 is:**Figure 2. Phylogenetic tree of gibbons and outgroup primates based on the mtDNA concatenated dataset.** The phylogenetic relationships among gibbons and six outgroup primates were inferred from the mtDNA concatenated dataset, including three partitioned sets: ribosomal RNA, transfer RNA and protein-coding gene. The maximum likelihood (ML) and Bayesian methods were used to reconstruct phylogenetic trees. Both analyses produced the same topology and their support values are indicated by circles on the nodes of the Bayesian tree shown here. Individuals used in the estimation of divergence times are marked with an asterisk.

The corrected Table 1 can be seen here: 

**Figure:**
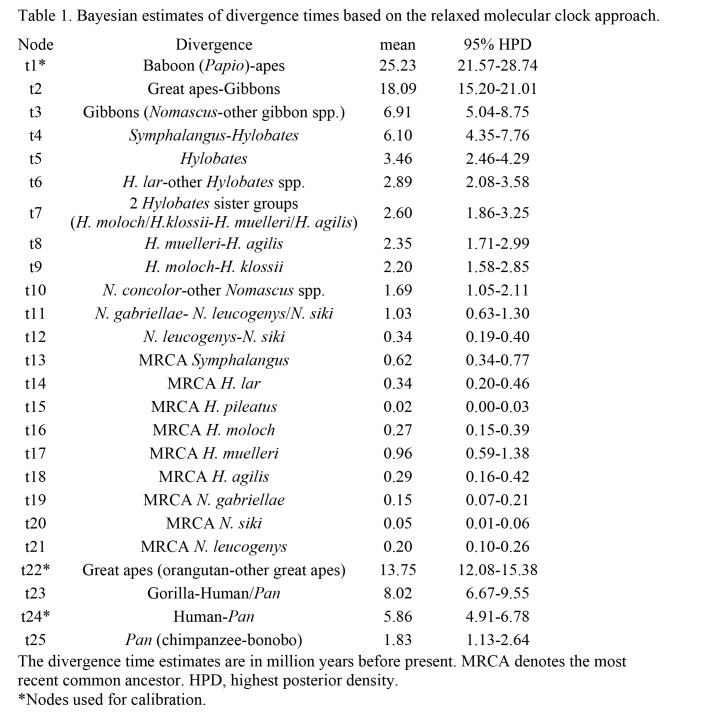


The corrected Table 4 can be seen here: 

**Figure:**